# Uncovering distinct protein-network topologies in heterogeneous cell populations

**DOI:** 10.1186/s12918-015-0170-2

**Published:** 2015-06-04

**Authors:** Jakob Wieczorek, Rahuman S Malik-Sheriff, Yessica Fermin, Hernán E Grecco, Eli Zamir, Katja Ickstadt

**Affiliations:** Faculty of Statistics, TU Dortmund University, Dortmund, Germany; Department of Systemic Cell Biology, Max-Planck Institute of Molecular Physiology, Dortmund, Germany; Present address: European Molecular Biology Laboratory, European Bioinformatics Institute (EMBL-EBI), Hinxton, Cambridge, UK; Present address: MRC Clinical Sciences Centre, Imperial College London, London, UK; Present address: Department of Physics, FCEN, University of Buenos Aires and IFIBA, CONICET, Buenos Aires, Argentina

**Keywords:** Bayesian analysis, Cluster analysis, Intercellular variability, Network analysis, Protein networks, Reverse engineering, Unmixing

## Abstract

**Background:**

Cell biology research is fundamentally limited by the number of intracellular components, particularly proteins, that can be co-measured in the same cell. Therefore, cell-to-cell heterogeneity in unmeasured proteins can lead to completely different observed relations between the same measured proteins. Attempts to infer such relations in a heterogeneous cell population can yield uninformative average relations if only one underlying biochemical network is assumed. To address this, we developed a method that recursively couples an iterative unmixing process with a Bayesian analysis of each unmixed subpopulation.

**Results:**

Our approach enables to identify the number of distinct cell subpopulations, unmix their corresponding observations and resolve the network structure of each subpopulation. Using simulations of the MAPK pathway upon EGF and NGF stimulations we assess the performance of the method. We demonstrate that the presented method can identify better than clustering approaches the number of subpopulations within a mixture of observations, thus resolving correctly the statistical relations between the proteins.

**Conclusions:**

Coupling the unmixing of multiplexed observations with the inference of statistical relations between the measured parameters is essential for the success of both of these processes. Here we present a conceptual and algorithmic solution to achieve such coupling and hence to analyze data obtained from a natural mixture of cell populations. As the technologies and necessity for multiplexed measurements are rising in the systems biology era, this work addresses an important current challenge in the analysis of the derived data.

**Electronic supplementary material:**

The online version of this article (doi:10.1186/s12918-015-0170-2) contains supplementary material, which is available to authorized users.

## Background

**Fig. 1:**
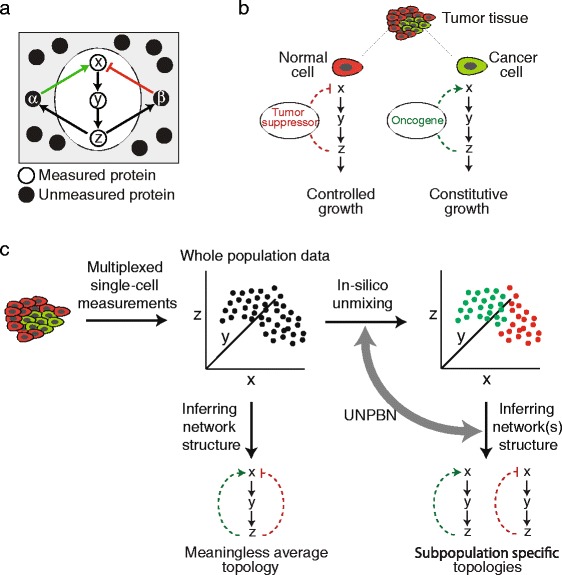
UNPBN addresses the challenge of studying intracellular protein networks caused by unmeasured proteins and inter-cellular heterogeneity. **a** A biochemical system for which three proteins (*x*, *y*, *z*) are being measured in the same cell while the other proteins are unmeasured. Note that the effects of *z* on *x* are mediated by unmeasured proteins (*α* and *β*). **b** Depending on the level and state of these unmeasured proteins, the measured causality between *x* and *z* can differ qualitatively between cells. For example, normal and cancer cells have different activity levels of oncogenes and tumor suppressors which here lead to a negative or a positive causal effect of *z* on *x*, respectively, thereby to a controlled growth or a constitutive growth. **c** Left, multiparametric high-throughput single-cell measurements (e.g., flow-cytometry) of a heterogenous sample of cells (e.g., cancer and normal cells). Middle, attempts to statistically infer a single set of relations (here, causal topology) between the measured proteins fail because there are two distinct subpopulations having two distinct sets of relations. At the same time, it is also impossible to identify the two distinct subpopulations as two distinct proximity-based clusters. Right, UNPBN performs unmixing and inference of statistical relations as one process, thus finds the set of sets-of-relations (network topologies) that explains best the observations

In order to understand how a protein network gives rise to a cellular function it is essential to quantify the states of the involved proteins and their causal relations. However, it is actually not possible to strictly define out of the proteome the subset of all proteins which are involved in a certain cellular process since these will always have interactions with proteins not included in this subset. In spite of major advances in proteomic [1, 2] and cytometric [3–7] methods, quantification of the levels and post-translational modifications of all proteins of the proteome in the same cell is still beyond reach. Therefore, we fundamentally cannot observe the whole system at once (i.e in the same cell) but only a small part of it (Fig. 1a) [8]. This limit, by itself, could have been overcome by looking at different parts of the system in different cells and building a model of the whole system step by step. However, such a strategy is fundamentally hampered by natural cell-to-cell variability which makes the integration of information highly challenging. Several studies have addressed the challenge of network reconstruction in the presence of intrinsic and extrinsic noise [9] around one prototypic network structure [10–12]. However, in many physiological cases the cell-to-cell variance is not only due to noise around one cellular state but also due to subpopulations which are in qualitatively distinct types of states. Such qualitative variabilities within the same cell population are generated by epigenetic commitment of cells to different fates (e.g., proliferation versus differentiation) as well as by genetic alterations (Fig. 1b) as in cancer [13, 14]. In many cases the distinct cell subpopulations are spatially intermixed and therefore are harvested together and co-measured within the same sample (e.g., by flow-cytometry). In such cases, causal relations and correlations between measured proteins can be qualitatively different in different cells if they are mediated by non-measured proteins which have different states at each subpopulation. Therefore, integration of observations over the cell population toward one model would be invalid and will yield uninformative average relations (Fig. 1c, middle). Ultimately, in order to solve this fundamental problem one should identify the number of qualitatively different subpopulations in the data, thus unmix the cells in-silico and resolve separately for each subpopulation the relations between the measured proteins. A recent work suggested to use a mixture model to unravel subpopulations in biochemical systems based on ordinary differential equations and prior knowledge about the number of subpopulations as well as about kinetic constants underlying the differences between them [15]. In this work we developed a Bayesian method for achieving this goal without such prior knowledge.

To unmix observations of cells from different subpopulations, we are taking advantage of the high-dimensionality of the observations, as typically obtained from cell-based high-content measurements such as flow-cytometry [3, 13, 16, 17], mass-cytometry [4, 5] and toponome imaging [6, 7]. Within each subpopulation, stochastic cell-to-cell variability in protein expression levels gives rise to high-dimensional probability distributions with the same dimensionality as the number of biochemical species (e.g., proteins) measured in each cell. To this extent, network inference approaches, like Gaussian Bayesian networks (GBN) [18–20], to resolve a single statistical model that fits best the data, have been already developed [21–23]. In this work we use as a basis our previously described nonparametric Bayesian network analysis (NPBN, [21]) and expand it to allow for different network structures in a mixture of different cell subpopulations (Fig. 1c, right). In this method, termed hereafter unmixing-via-NPBN (UNPBN), a flexible number of Gaussian Bayesian networks is being fitted to the data and thereby iteratively identifying the number of distinct subpopulations, unmixing the observations and resolving the statistical model for each subpopulation. As a model system to assess and demonstrate our method we simulated the canonical MAPK Raf-Mek-Erk kinases cascade in the context of PC12 cells stimulated by either epidermal growth factor (EGF) or nerve growth factor (NGF) [24]. We show that our method identifies better than common clustering approaches the presence of two subpopulations within a mixture of EGF and NGF stimulated PC12 cells based on the levels of active Raf, Mek and Erk in each cell. This enabled to resolve correctly the statistical relations between Raf, Mek and Erk in each subpopulation.

## Methods

### Simulation

The EGF and NGF signaling network was simulated based on a previously described model [24]. The SBML format of this model (BIOMD0000000049, www.ebi.ac.uk, retrieval date Oct. 24, 2011) was imported into the Matlab Symbiology platform to simulate the dynamics of the signaling network using *ode15s* (stiff/NDF) solver. To introduce intra-subpopulation cell-to-cell variability (termed herein noise), for each run of the simulation we sampled the values for the total Raf, Mek and Erk levels from a Normal distribution *N*(*μ*,*σ*) around the respective initial concentration for a given set of fractional deviation from the mean (*σ*=*μ*·*fd*, where *fd*∈{0.1,0.2,0.3,0.4,0.5,0.6,0.7}). The values of *fd* represent here the degree of stochastic variance in the expression levels of Raf, Mek and Erk. Simulations were repeated 175 times with random sampling of total Raf, Mek and Erk levels to generate the data for each cell subpopulation. In each individual simulation repeat, the response of the network to EGF or NGF was simulated for 600 seconds after stimulation and the levels of c-Raf-Ras-GTP (hereafter referred as pRaf, reflecting the consequently activated Raf), ppMek and ppErk (the active, double phosphorylated, forms of Mek and Erk, respectively) were sampled every 1 minute as the observed parameters for the unmixing analysis. Mixtures containing two distinct cell subpopoulations were generated by mixing an equal number, unless indicated otherwise, of simulated observations obtained upon EGF and NGF stimulations. Mixtures containing four distinct cell subpopoulations were generated by altering the parameter in the SBML model corresponding to the catalytic activity (*k*_*cat*_) of Mek (J136) from its wild-type (Mek ^*wt*^) value (*k*_*cat*_ = 0.15 *s*^−1^) to a value depicting a mutant Mek (Mek ^*mut*^) with a lower activity (*k*_*cat*_ = 0.015 *s*^−1^). Thus, by having two different stimulations and two different levels of Mek activity, observations of four distinct cell subpopulations were generated: EGF-Mek ^*wt*^, EGF-Mek ^*mut*^, NGF-Mek ^*wt*^ and NGF-Mek ^*mut*^ (Additional file 1a-d).

### UNPBN

Methodologically, UNPBN is based on the nonparametric Bayesian networks (NPBN) approach [21]. It allows to avoid the assumption of underlying Gaussian distributions for the data and to find networks with nonlinear relations between the nodes. The UNPBN method combines a nonparametric mixture model incorporating the Dirichlet process [21, 25] and an allocation sampler [26, 27]. Prior to the description of the UNPBN approach a short introduction of GBNs [28] is provided here, as they are a basis of the presented method. We define the data *X*, consisting of *n* observations of a system/network with *d* species/nodes ($X \in \mathbb {R}^{n \times d}$), such that *x*_*j*_ represents an *n*-dimensional vector containing the observed concentrations of the *j* species (*j*=1,…,*d*). In the Bayesian networks approach the relations between the nodes in a graph $\mathcal {G}$ are modeled as conditional probability distributions (CPDs) *p*. If the CPDs for all nodes in $\mathcal {G}$ are given by Normal distributions of the form $x_{j}|{pa}_{\mathcal {G}}(x_{j})\sim N(\mu _{j}+\underset {\mathcal {K}_{j}}{\sum }\beta _{j,k} (x_{k}-\mu _{k}), {\sigma _{j}^{2}})$, where ${pa}_{\mathcal {G}}(x_{j})$ denotes the parents of node *x*_*j*_, $\mathcal {K}_{j}=\{k|x_{k} \in {pa}_{\mathcal {G}}(x_{j})\}$, the *μ*_*j*_ and ${\sigma _{j}^{2}}$ are the unconditional means and variances of *x*_*j*_, and *β*_*j*,*k*_ are real-valued coefficients determining the influence of *x*_*k*_ on *x*_*j*_, and, if in addition, $\mathcal {G}$ is a directed acyclic graph (DAG) then the pair $(p,\mathcal {G})$ is called a GBN. The network structure is inferred using Gaussian distributions with a Normal-Wishart prior [20]. The estimation of $\mathcal {G}$ is embedded in a Markov Chain Monte Carlo (MCMC) framework, conducted by maximizing the sampling distribution of the sampled graph

$$\begin{array}{@{}rcl@{}}  L\left(\mathcal{G}| X\right) &=& \prod_{j=1}^{d} \int L\left({\sigma_{j}^{2}}, \beta_{j}| X^{\left(\{j\} \cup\mathcal{K}_{j}\right)}\right)p\left({\sigma_{j}^{2}}, \beta_{j}\right)d\sigma_{j} d \beta_{j}, \end{array} $$

with *β*_*j*_=(*β*_*j*,1_,…,*β*_*j*,*j*−1_),(*β*_2_,…,*β*_*d*_)=*B* and $X^{(\mathcal {J})}$ denotes the columns of *X* with indices in $\mathcal {J}$. The MCMC algorithm uses so called single edge operations [29].

UNPBN generalizes the GBN approach as it is based on flexible nonparametric Bayesian mixture models for networks [21] which in turn combine different GBNs for different subsets of the data. The mixture is taken with respect to all parameters $(\mu, \sigma, B, \mathcal {G})$. The model for the data can be written as $p(x) = \int p(x|\mu,\sigma, B,\mathcal {G})dP(\mu, \sigma, B, \mathcal {G})$ with *μ* and *σ* vectors of the unconditional means *μ*_*j*_ and variances ${\sigma _{j}^{2}}$, respectively. The discrete mixing measure *P* is distributed according to $\mathbb {P}$, a random probability measure, and $p(x|\mu,\sigma, B,\mathcal {G})$ is a multivariate Normal distribution with a conditional independence structure compatible with $\mathcal {G}$. According to the discrete nature of *P*, support points $\mu _{h},\sigma _{h}, B_{h}, \mathcal {G}_{h}$ and probabilities *w*_*h*_, the mixture can be written as

$$\begin{array}{@{}rcl@{}} p(x) = \sum_{h=1}^{N} w_{h} \ p(x| \mu_{h}, \sigma_{h}, B_{h}, \mathcal{G}_{h}). \end{array} $$

The prior distribution of the mixing weights *w*_*h*_ is assigned by *P* and the prior for $\mu _{h},\sigma _{h}, B_{h}, \mathcal {G}_{h}$ is given by the base measure *P*_0_ of $\mathbb {P}$ for all *h*. The *N* different mixture components *h* can be interpreted as subpopulations in the data set. Accordingly, here such subpopulations are referred to as components. The assignment of each data point to its corresponding component is described by the allocation vector *l*=(*l*_1_,…,*l*_*n*_)^′^ [26].

The network structure $\mathcal {G}$ and the allocation vector *l* are the main focus of our UNPBN procedure. The remaining parameters *μ*_*h*_, *σ*_*h*_ and *B*_*h*_ are integrated out and the MCMC algorithm iterates by updating the DAG $\mathcal {G}$, the number of components *N* and the latent allocation vector *l*, leading to the posterior distribution

$$\begin{array}{@{}rcl@{}} p(l,\mathcal{G},N|X)= \prod_{h=1}^{N} L(\mathcal{G}| X_{(\mathcal{I}_{h})})p_{N}(m)p(N)p(\mathcal{G}), \end{array} $$

where $L(\mathcal {G}| X)=\int L(\sigma, B|X)p(\sigma, B)d{\sigma }d{B}$ is the marginal sampling distribution for $\mathcal {G}$, *p*_*N*_(*m*) is a probability distribution on the space of allocation vectors, *p*(*N*) is the distribution of the number of components and $\mathcal {I}_{h}=\{i \in \{1,\:\ldots,n\}|l_{i}=h\}$ and $X_{(\mathcal {I})}$ denotes the rows of *X* with indices in $\mathcal {I}$.

In our UNPBN analysis a prior is needed for $ \theta _{h}=(\mu _{h}, \sigma _{h}, B_{h}, \mathcal {G}_{h})$ and for *w*_1_,…,*w*_*N*_. For $\mathcal {G}_{h}$ the prior which was used is uniform over the cardinality of the parent sets [30], for *σ*_*h*_ and *B*_*h*_ we employed the Normal-Wishart prior distribution with the identity matrix as the precision matrix and *d*+2 degrees of freedom. The mean vector of the multivariate Normal distribution (*μ*_*h*_) was chosen as a vector of zeros. For *N* we used a Poisson distribution with parameter *λ*=1 and the *w*_*h*_ were obtained from a Dirichlet distribution with parameter vector (*α*,…,*α*) with *α*=1. Further details for the sampling distribution, posterior distribution and the MCMC sampling scheme were discussed in previous publications [21, 26, 31]. The approach is implemented in Matlab (R2009b, The MathWorks Inc., Natick, Massachusetts). The presented results are obtained from MCMC runs with 2.8·10^6^ iterations with a thinning of 350 and a burn in of 1.4·10^6^ iterations for networks with two subpopulations and from runs with 5·10^6^ iterations with a thinning of 500 and a burn in of 2·10^6^ iterations for networks with four subpopulations.

### Postprocessing of graphs

Although it is possible to use the output from the UNPBN analysis directly, for example to choose the most frequent DAG or allocation vector as a representative, it is preferable to perform an additional postprocessing step that takes into account all MCMC samples and improves the results considerably. The inferred graphs in the iterations of the MCMC simulation are stored in the form of adjacency matrices. Such a matrix *A* consists of the elements *a*_*ij*_(*i*,*j*=1,…,*d*), *a*_*ij*_=1 if nodes *i* and *j* are conditionally dependent (an arrow leading from node *i* to node *j*) and *a*_*ij*_=0 if nodes *i* and *j* are conditionally independent (no arrow between them) or if *i*=*j*. For each pair of nodes the MCMC output of the UNPBN analysis can be summarized by the posterior edge probability ${pep}_{i j} = \sum _{s=1}^{r} a_{i j}^{s} / r$ where *s* is the index of the *r* iteration steps in the MCMC simulation. The resulting *pep* number ranges from 0 (i.e., strong evidence for the absence of a connection) to 1 (i.e., strong evidence for a connection between the corresponding nodes). These *pep* values are used in Fig. 5 for the presentation of the obtained results.

**Fig. 2:**
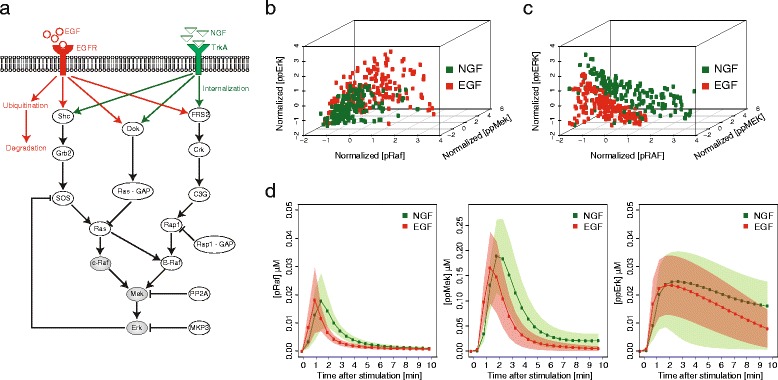
Simulations of the EGF and NGF signaling network in PC12 cells. **a** The simulated protein network, as previously described [24]. The elements (proteins, interactions and processes) that are unique to either EGF or NGF are colored red and green, respectively. The components that we later consider as the measured components of that system (i.e. Raf, Mek and Erk) are colored grey. **b** A 3-dimensional scatter plot showing the normalized levels of active (i.e. phosphorylated) Raf (pRaf), Mek (ppMek) and Erk (ppErk) versus each other at 2 minutes after NGF (green) or EGF (red) stimulation with noise level of 0.7. **c** The same as **b** but for 9 minutes after stimulation. **d** Time profiles of pRaf, ppMek and ppErk levels as a function of time after NGF (green) or EGF (red) stimulations, without noise (solid line) or with 0.7 noise level (green and red shadows)

**Fig. 3:**
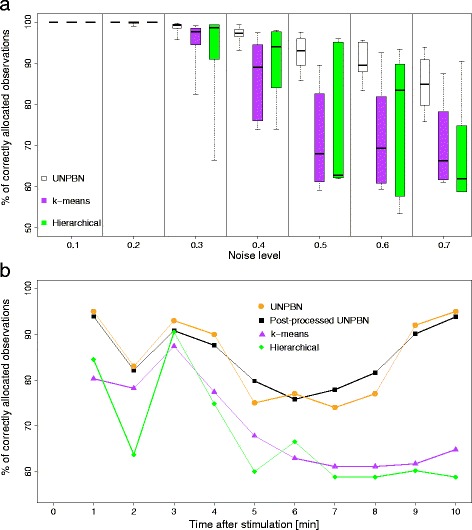
Unmixing observations of a mixed cell population by UNPBN in comparison to clustering approaches. **a** Mixtures of observations of EGF and NGF stimulated cells with different noise levels were generated as described. Observations were sampled at one-minute intervals for 10 minutes after stimulation. For each noise level and sampled time point, observations were unmixed using UNPBN, k-means clustering (with *k*=2) and hierarchical clustering (taking the final two clusters). The percentages of correctly allocated observations, averaged over all time points, are indicated by boxplots for the different methods as a function of the noise level (line within the box, the median; box, the 0.25 and 0.75 quartiles; whiskers, the largest and smallest data points which are still within the interval of 1.5 times the interquartile range from the box). **b** Comparison of the unmixing accuracy with noise level 0.7 along the different sampled time points, as achieved by UNPBN, post-processed UNPBN limited to two components, k-means (with *k*=2) and hierarchical clustering (taking the final two clusters)

**Fig. 4:**
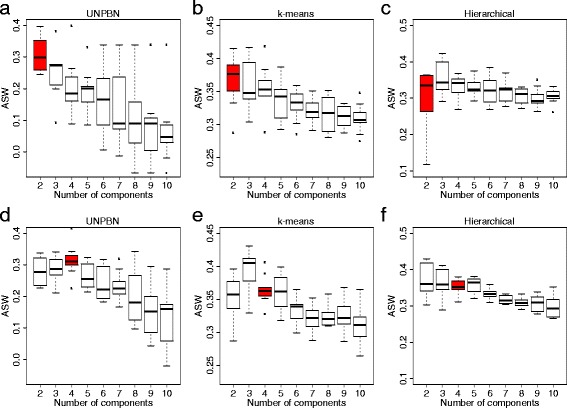
The success in identifying the correct number of distinct cell subpopoulations (i.e. components) in a mixture by UNBPN in comparison to clustering approaches. **a** A boxplot showing the ASW versus the tested number of components obtained by UNBPN analysis (here constrained in the postprocessing step to the imposed number of components) of a mixture of two subpopulations (EGF and NGF stimulated cells). The boxplot indicates the median (line within the box), the 0.25 and 0.75 quartiles (box), margined by the largest and smallest data points which are still within the interval of 1.5 times the interquartile range from the box (whiskers), and the outliers (dots) obtained from pooled values over all time points with noise level of 0.5. **b** and **c**, the same as in **a** but for ASW obtained following k-means clustering and hierarchical clustering, respectively. **d**, **e** and **f**, the same as the corresponding **a**, **b** and **c**, but for a mixture of 4 subpopulations: EGF-Mek ^*wt*^, EGF-Mek ^*mut*^, NGF-Mek ^*wt*^ and NGF-Mek ^*mut*^ (Additional file 1a-d). It should be noted that silhouette widths are incomparable between different clustering approaches. However, silhouette widths are comparable between different parameters of the same clustering approach and thereby indicate the identified number of distinct subpopoulations as the one providing the largest ASW

**Fig. 5:**
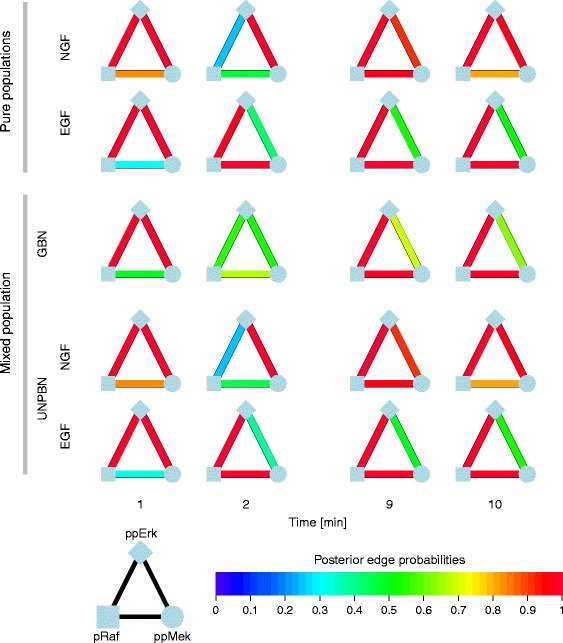
Recovering correctly the edge probabilities network between pRaf, ppMek and ppErk for each cell subpopulation in a mixture by UNPBN. The triangles show, color encoded, the posterior edge probabilities between pRaf, ppMek and ppErk at different time points after stimulation as derived by UNPBN analysis of pure NGF-stimulated or EGF-stimulated cell populations (top two rows) with noise level of 0.2 and of a mixture of these populations by GBN versus UNPBN (bottom three rows). Note, for the pure subpopulations, the changes in the edge probabilities between the same components at different time points and upon the different stimulations. Analyzing a mixture of NGF-stimulated or EGF-stimulated cells without unmixing yields uninformative average edge probabilities, not representing any of the distinct two subpopulations. Employing UNPBN recovers precisely the pure edge probabilities for each of the two subpopulations. For clarity, we show here the edge probabilities for 4 time points after stimulation that illustrate the need for unmixing

### Postprocessing of allocations

For the allocation vector, however, it is not possible to summarize the sampled vectors in the same way as for the edges, because of the so called label switching problem. During the sampling procedure the labels of the components change randomly, so that if two allocation vectors are compared it is not clear if a particular observation has been allocated to a different component or if the label of the component has changed. We employed a method based on maximizing the adjusted *Rand index* that bypasses this obstacle and that combines the allocation vectors of each MCMC iteration into one single vector [32]. This method is implemented in R [33] in the package ’mcclust’ and was used in cases where it was necessary to fix the number of components to a particular value (Fig. 4). In cases where the analysis is focused on the unmixing performance of UNPBN (Fig. 3), the sampled allocation vectors are evaluated regarding the homogeneity of the resulting components. For each entry $l_{i}^{s,h}$ in the allocation vector sampled in iteration *s*, in each component *h*, the true component is determined by comparison with the simulation setting. Based on this, componentwise, observations originating from the same true component are considered as allocated correctly, (the indicator function $I\left (l_{i}^{s,h}\right)$ is set to 1) while the remaining observations in that component are considered as wrongly allocated ($I \left (l_{i}^{s,h}\right)=0$). The percentage of correctly allocated observations (*pco*) for a particular UNPBN outcome is derived by

$$\begin{array}{@{}rcl@{}} \textit{pco} = \frac{1}{r} \sum^{r}_{s=1} \frac{1}{N^{s}} \sum^{N^{s}}_{h=1} \frac{1}{{n_{h}^{s}}} \sum^{{n_{h}^{s}}}_{i=1} I \left(l_{i}^{s,h}\right) \cdot 100 \end{array} $$

with *r* considered MCMC iterations, size ${n_{h}^{s}}$ of component *h* in iteration *s* and *N*^*s*^ number of components in the allocation vector in iteration *s*.

### Cluster analyses

In order to compare UNPBN with clustering methods, k-means and hierarchical clustering were used in this work. The k-means clustering method finds the partition that divides the data to *n* clusters (where *n* is given by the user) such that the sum distances of all observations to the corresponding cluster mean is minimized [34]. The k-means cluster analysis was performed in Matlab, using the function “kmeans” with the distance parameter being set to squared Euclidean distance. To obtain stable results, the clustering was repeated 500 times with randomly chosen different starting points. Hierarchical clustering is an agglomerative procedure which merges in each step the two closest objects, repeatedly till the whole data set is in one single cluster. The hierarchical clustering was performed in Matlab using the functions “pdist”, with the distance parameter being set to Euclidean, followed by “linkage”, with the method parameter being set to inner squared distance (“ward”, [35]).

### Silhouette analysis

We used the average silhouette width (ASW) [36], to assess the quality of a given clustering and to compare the results of clusterings with different parameter settings. For a given clustering result, the silhouette value is calculated as

$$ sil(x_{i}) = \frac{b(x_{i})-a(x_{i})}{\max\{a(x_{i}), b(x_{i}) \}} . $$

For each observation, *x*_*i*_, *a*(*x*_*i*_) is the average dissimilarity between *x*_*i*_ and all other data points within the same cluster, and *b*(*x*_*i*_) is the smallest average dissimilarity between *x*_*i*_ and the data points in the remaining clusters, calculated for each cluster separately. Any measure of dissimilarity can be used, but distance measures are the most common. In this work the Euclidean distance was employed. The silhouette value is ranging between -1 and 1. Negative values indicate that a particular observation will fit better in another cluster, so it has been matched wrongly and the quality of the clustering result can be improved. High positive values indicate a good clustering result. The ASW is computed by averaging all *sil*(*x*_*i*_) values, thus it provides an overall evaluation of the regarded clustering. While ASW enables to compare clustering performed with the same method with different parameters, ASW values are not comparable between different clustering methods.

## Results

### Simulation of inter and intra cell-population variabilities

In order to evaluate the performance of the method we simulated the MAPK module in PC12 cells using a previously described model [24]. This model captures the different temporal profiles of Erk activation upon EGF and NGF stimulation, attributing it to the differential activation and dynamics of Ras and Rap (Fig. 2a) [24, 37]. Both stimulations activate via Sos and Ras the upstream kinase, Raf and thereby the whole MAPK cascade. However, each stimulation has a different effect on other proteins which affect the MAPK module and its dynamics. In EGF stimulation, Erk inhibits Sos and thereby forms a negative feedback loop leading to a transient Erk activation which encodes a proliferation signal. In NGF stimulation this negative feedback is overcome by a nested positive feedback loop [38] formed due to the activation of PKC *δ* which phosphorylates RKIP and thus leads to its release from Raf and thereby enabling Raf activation by Erk. The model used here considers another difference attributed to a sustained activation of another activator of the MAPK cascade, Rap1, by NGF but not by EGF [24, 37]. Thus, NGF leads to a sustained Erk activation, encoding a signal for differentiation. As a source for inter-population variability, we simulated the dynamics of the complete network upon either EGF or NGF stimulation. For the aim of this work, we based our analyses on snapshots of the simulation, and, in turn, analyzed each time point independently. As a source of intra-population variability (hereafter referred to as noise), we added stochastic noise in total protein levels mimicking natural cell-to-cell variance in protein expression (see Methods). However, unlike instrumental noise that only affects the readout, noise in expression levels affects the system itself. Thus, although the introduced noise was generated as Gaussian, its propagation through the system generates asymmetric high-order patterns shaped by the topology of the network (Fig. 2b,c).

In the absence of noise, the levels of phosphorylated (thus activated) Raf, Mek and Erk follow the expected profiles, exhibiting a clear difference between EGF or NGF stimulations (Fig. 2d, red and green solid lines). With intra-population variance, the profiles get broader and overlap between the two stimulations (Fig. 2d, red and green shadows), making it difficult to allocate individual observations to the corresponding stimulation (as for example at 2 minutes after stimulation, Fig. 2b). To impose the fundamental experimental limit of observing only part of the system, for the subsequent analysis we considered an observation to be the triplet formed by the concentrations of phosphorylated species of Raf, Mek and Erk per cell, ignoring all other information. Finally, to generate heterogeneous cell-populations, we mixed observations randomly selected in equal amounts from the EGF and NGF datasets.

### UNPBN unmixes observations of distinct subpopulations

We first wanted to test whether UNPBN can classify correctly observations coming from distinct cell subpopulations. We applied UNPBN on mixed cell populations having different levels of noise and counted the observations correctly allocated to the EGF and NGF stimulated subpopulations (Fig. 3a). For noise levels of 0.1, 0.5 and 0.7, around 100 %, 93 % and 85 % of the observations are correctly allocated, respectively (Fig. 3a). To assess the accuracies of UNPBN, we compared them to those achieved by two widely used clustering approaches - hierarchical clustering and k-means clustering. When the noise is low (0.1 and 0.3), the two subpopulations are well separated by all methods (Fig. 3a). As expected, the performance of all three methods is negatively affected when the noise level is increased. However, the UNPBN considerably outperforms the other reference methods for all noise levels above 0.2 (Fig. 3a). If the relative abundance of the two subpopulations is 1:9, all methods classify about equally well for a low noise level (0.2), while for a high noise level (0.7) UNPBN classifies as good as k-means but better than hierarchical clustering (Additional file 2).

We next focused on the high noise level of 0.7 and compared the performance of the methods as a function of time after stimulation (Fig. 3b). Along the different time points the performance of all methods varies, reflecting a changing difficulty to identify the two subpopulations based on the levels of pRaf, ppMek and ppErk. UNPBN constantly outperforms the clustering methods in all the time points and is more robust with its performance level (Fig. 3b). Furthermore, the performances of the two clustering methods along the time points have a similar profile, which differs from the profile of UNPBN (Fig. 3b, time points 3-10 minutes). These results are consistent with the fact that, unlike the clustering approaches, UNPBN uses high-order patterns, rather than merely distances between observations. This additional information is shown here to be indeed valuable for the ability to unmix subpopulations based on high-dimensional observations.

### UNPBN identifies the number of subpopulations in a mixture

In many cases, when a sample of cells is derived it is unknown a priori how many distinct subpopulations it contains. Therefore, a comprehensive unmixing approach should also be able to identify the number of subpopulations without such a priori knowledge. Indeed, while the clustering approaches were guided to search for two subpopulations, UNPBN was not given this information but found it independently (Fig. 3b). Moreover, the performance of UNPBN does not change significantly if it is forced to identify exactly two subpopulations, indicating the ability of UNPBN to correctly determine by itself the number of distinct subpopulations in a mixture (Fig. 3b).

In order to compare the capability of the different methods to identify the number of subpopulations we used the ASW to determine the quality of the clusters and thereby the number of clusters (i.e. subpopulations) in the data as could be inferred by each method [36]. The ASW of a cluster is a measure of how tightly grouped are the data points in the cluster, such that larger ASW values denote tighter clusters. For a cell population containing two subpopulations (EGF and NGF stimulated cells, Additional file 1a,b) we calculated the ASW as a function of the number of clusters derived by UNPBN (here constraints by postprocessing yield an imposed number of components), k-means and hierarchical clustering (Fig. 4a-c). For UNPBN and k-means clustering, the maximal ASW is found when the number of clusters is 2, the actual number of subpopulations in the data (Fig. 4b,c, red bars). However only in UNPBN there is a significant and robust difference with the other cluster sizes, while with k-means clustering the ASWs obtained for 2 and 3 clusters are not robustly separable. With hierarchical clustering the performance is further worse since ASWs obtained for 3 and 4 clusters are comparable, or even higher than those obtained for 2 clusters (Fig. 4c). UNPBN successfully identified the number of subpopulations also if their relative abundance was significantly different (1:9, see Additional file 3).

We next tested the performance of the method with a more complex mixture of cells containing four distinct subpopulations. To simulate these subpopulations, the catalytic rate constant, *k*_*cat*_, of Mek in the model was changed, mimicking a wild-type Mek (Mek ^*wt*^) and a mutant Mek (Mek ^*mut*^) that phosphorylate Erk at different rates. Thus, together with the two different stimulations, EGF and NGF, four distinct subpopulations were generated, denoted by EGF-Mek ^*wt*^, EGF-Mek ^*mut*^, NGF-Mek ^*wt*^ and NGF-Mek ^*mut*^ (Additional file 1a-d). UNPBN correctly identified that the data contains four distinct subpopulations, in contrast to k-means and hierarchical clustering (Fig. 4d-f).

### UNPBN uncovers distinct topologies for distinct subpopulations

The causal relations between the components of a system are constant, since the set of biochemical reactions and constants that describe the whole system remains constant. However, in practice, only part of the components of a system can be co-measured and therefore the reaction constants become apparent constants that depend on the unmeasured components. Here we intentionally simulated the fundamental limit of looking on only a small part of a system. Therefore we expected the apparent strength of the causal connection between pRaf, ppMek and ppErk, as reflected by the undirected posterior edge probabilities among them, to change as a function of the stimulation and time. Indeed, when we analyzed separately EGF and NGF stimulated cells we observed different posterior edge probabilities between the two treatments, as well as within each treatment at the different time points (Fig. 5). When analyzing the mixed population with a standard GBN approach (i.e. without the possibility of unmixing) [39], we obtained posterior edge probabilities exhibiting, in general, an average behavior of the two subpopulations. Naturally, these average values become meaningless when the two subpopulations exhibit very different posterior edge probabilities (e.g., at 2 minutes, Fig. 5). In contrast, when analyzing the mixed population with UNPBN (i.e., with unmixing), the unmixing step enabled to uncover the true network of posterior edge probabilities for each stimulation and at each time point (Fig. 5). This also demonstrates that the performance of the unmixing process (Fig. 3) was sufficiently good to enable correct inference of protein-protein relations in each subpopulation. Since more than one DAG may represent exactly the same set of conditional independence relationships [40], given static data without perturbations it is more reliable to infer the causal strengths between proteins, regardless of the direction of these causalities. Extending the UNPBN approach to dynamic data, or using perturbation data or adding prior information, will further facilitate the inference of directionality in the causal relations for each cell subpopulation.

## Discussion

In the era of systems biology, single-cell measurement techniques are rapidly expanding with respect to the number of cells that can be analyzed and the number of biochemical species that can be co-measured per cell. The approaches to explore these data have focused so far either on identifying different subpopulations of cells based on multiparametric proximities or on inferring the topology of statistical relations between the parameters for the population as a whole. However, the aim to reach each of these two goals in separate has fundamental problems. In one direction, ignoring the heterogeneity between cell subpopulations will lead to inferring a meaningless average topology of statistical relations of the population as a whole. In the other direction, since statistical relations are inferred from the correlation between the measured parameters, the identification of cell subpopulations based on multiparametric proximities inherently conflicts with the capability to resolve the topology of relations within each subpopulation. Furthermore, protein networks with distinct topologies can be at the same state (i.e., to have high multiparametric proximity) and protein networks with the same topology can be at different states (e.g., at different phases along an oscillatory response). Therefore, attempts to identify cell subpopulations based on multiparametric proximities may actually identify different cellular states but not different types of cells. The method presented here pioneers a comprehensive solution to these fundamental problems by performing the identification of cell subpopulations (i.e. unmixing) and the inference of statistical relations between the measured parameters in one joint analysis.

Intentionally, we used snapshot data of a dynamic process (the response of cells to EGF or NGF stimulations) and, respectively, the method we developed does not rely on temporal information nor intends to give a model description of the dynamic itself. Due to that, this method can be applied on the type of single-cell multiparametric measurements currently available such as multicolor flow-cytometry [3, 16], multiplexed mass cytometry [4, 5] and toponome imaging [6, 7]. The classification of the distinct subpopulations in cell populations sampled at different time points along an experiment can hint toward the dynamic behavior of each subpopulation. However, such traceability of subpopulations along the time points depends on how different is their relative abundance within the whole population and on the sampling rate in comparison to the timescale of the biological process. Advances in multicolor live cell imaging in combination with high-throughput automated microscopy gradually facilitate monitoring increasing numbers of parameters in individual live cells over many cells. The data obtained from such measurements will enable not only tracking the dynamics of the measured parameters in each cell subpopulation but also tracking them in individual cells. This kind of temporal information will help to further improve the identification of the distinct cell subpopulations and the inference of statistical relations between measured parameters in each subpoulation. As indicated by this work, it would be important also for the analysis of such live cell measurement data that unmixing and inference of protein-protein relations will be performed as one process.

The importance to recover single-cell phenotypes out of an uninformative average cell population behavior has been established and exemplified in many systems. Notably, in these examples there was only one measured parameter per cell, often the output of the system, and, therefore, the unmixing was straightforward. However, in order to obtain mechanistic insight into how a biochemical system works it is required to examine the protein network itself, and not only its output. For this, multiple parameters should be co-measured per cell to overcome uncorrelated cell-to-cell variability between these parameters (e.g., due to stochastic noise in expression levels as simulated in this work). We demonstrated here that in such a case unmixing cannot be achieved anymore using the proximity between the values of these parameters, while it can be successfully achieved using the high-order relations between them as captured by UNPBN. Importantly, UNPBN can be straightforwardly extended to incorporate prior knowledge about parts of the network in the individual subpopulations.

## Conclusions

Our results show that the coupling between unmixing of observations and inference of statistical relations is essential and effective. With respect to the unmixing, our method was capable to identify the number of qualitatively distinct subpopulations considerably better than multiparametric proximity based approaches (hierarchical clustering and k-means clustering). Consequently, the statistical relations in each unmixed subpopulation were also correctly recovered, while, without unmixing, uninformative average relations were inferred. As systems biology and personalized medicine are aiming toward reverse-engineering and re-engineering signaling networks, they are increasingly challenged by the inter-cellular variability and the large size of the relevant biochemical system. The work presented here offers a conceptual solution as well as an applicable statistical method to address this challenge.

## Additional files

Additional file 1
**The four distinct simulated topologies of the EGF and NGF signaling network used in Fig.** 4. (a) NGF-Mek ^*w**t*^: the wild-type network (see Methods) with NGF stimulation. (b) EGF-Mek ^*w**t*^: as in (a) but with EGF stimulation. (c) NGF-Mek ^*m**u**t*^: the wild-type network with NGF stimulation, beside that here the SBML model parameter corresponding to the *k*_*cat*_ of Mek (J136) is altered from its wild-type value (*k*_*cat*_ = 0.15 *s* ^−1^) to a value depicting a mutant Mek with a lower activity (*k*_*cat*_ = 0.015 *s* ^−1^), as indicates the thinner arrow from Mek to Erk. (d) EGF-Mek ^*m**u**t*^: as in (c) but with EGF stimulation.

Additional file 2
**Unmixing observations of cell subpopulations, mixed in a 1:9 ratio, by UNPBN in comparison to clustering approaches.** Mixtures of observations of EGF stimulated cells (90 %) and NGF stimulated cells (10 %) were generated with noise levels of 0.2 and 0.7. Observations were sampled at one-minute intervals for 10 minutes after stimulation. For each noise level and sampled time point, observations were unmixed using UNPBN, k-means clustering (with *k*=2) and hierarchical clustering (taking the final two clusters). The percentages of correctly allocated observations, averaged over all time points, are indicated by boxplots for the different methods for both noise levels (line within the box, the median; box, the 0.25 and 0.75 quartiles; whiskers, the largest and smallest data points which are still within the interval of 1.5 times the interquartile range from the box). (a) The percentages of correctly allocated observations of the EGF-stimulated subpopulation. (b) The percentages of correctly allocated observations of the NGF-stimulated subpopulation.

Additional file 3
**The success of UNPBN in identifying the correct number of cell subpopulations (i.e. components) mixed in a 1:9 ratio.** Mixtures of observations of EGF stimulated cells (90 %) and NGF stimulated cells (10 %) were generated with noise levels of 0.2 and 0.7. Observations were sampled at one-minute intervals for 10 minutes after stimulation. (a) A boxplot showing the ASW versus the tested number of components obtained by UNBPN analysis (here constrained in the postprocessing step to the imposed number of components). Each boxplot indicates the median (line within the box), the 0.25 and 0.75 quartiles (box), margined by the largest and smallest data points which are still within the interval of 1.5 times the interquartile range from the box (whiskers), and the outliers (dots) obtained from pooled values over all time points with a noise level of 0.2. (b) The same as (a) but with a noise level of 0.7.

## Abbreviations

ASW: Average silhouette width
DAG: Directed acyclic graph
EGF: Epidermal growth factor
Erk: Extracellular signal regulated kinase
GBN: Gaussian Bayesian network
MAPK: Mitogen-activated protein kinase
MCMC: Markov Chain Monte Carlo
Mek: MAPK/Erk kinase
NGF: Nerve growth factor
NPBN: Nonparametric Bayesian network analysis
PKC: Protein kinase C
UNPBN: Unmixing via NPBN
