# Supplementary figures and images for: Uncovering distinct protein-network topologies in heterogeneous cell populations
Source: BMC Syst Biol. 2015 Jun 4;9:24. doi: 10.1186/s12918-015-0170-2 (PMC4480582; doi:10.1186/s12918-015-0170-2)

**a****NGF-Mek<sup>wt</sup>**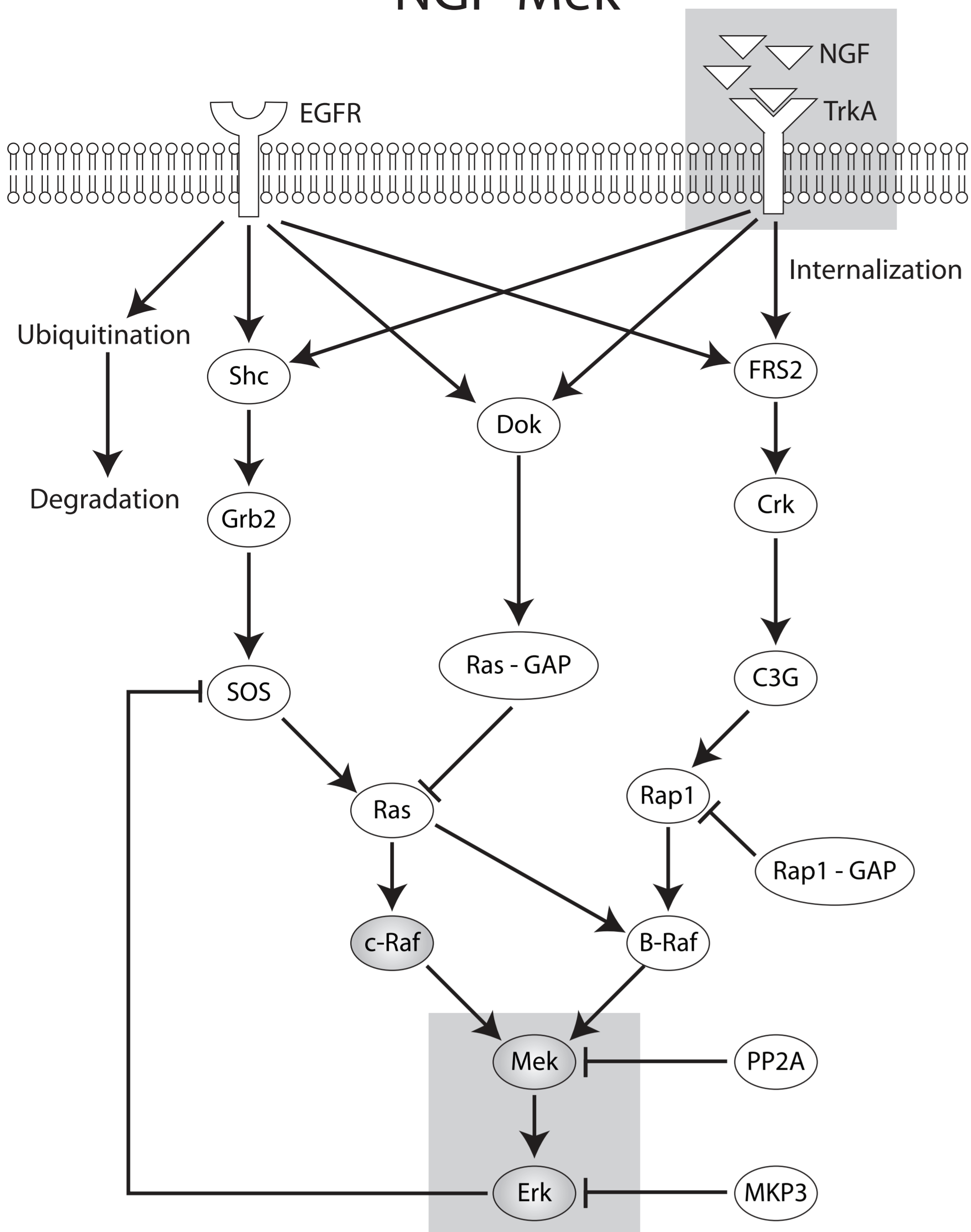**b****EGF-Mek<sup>wt</sup>**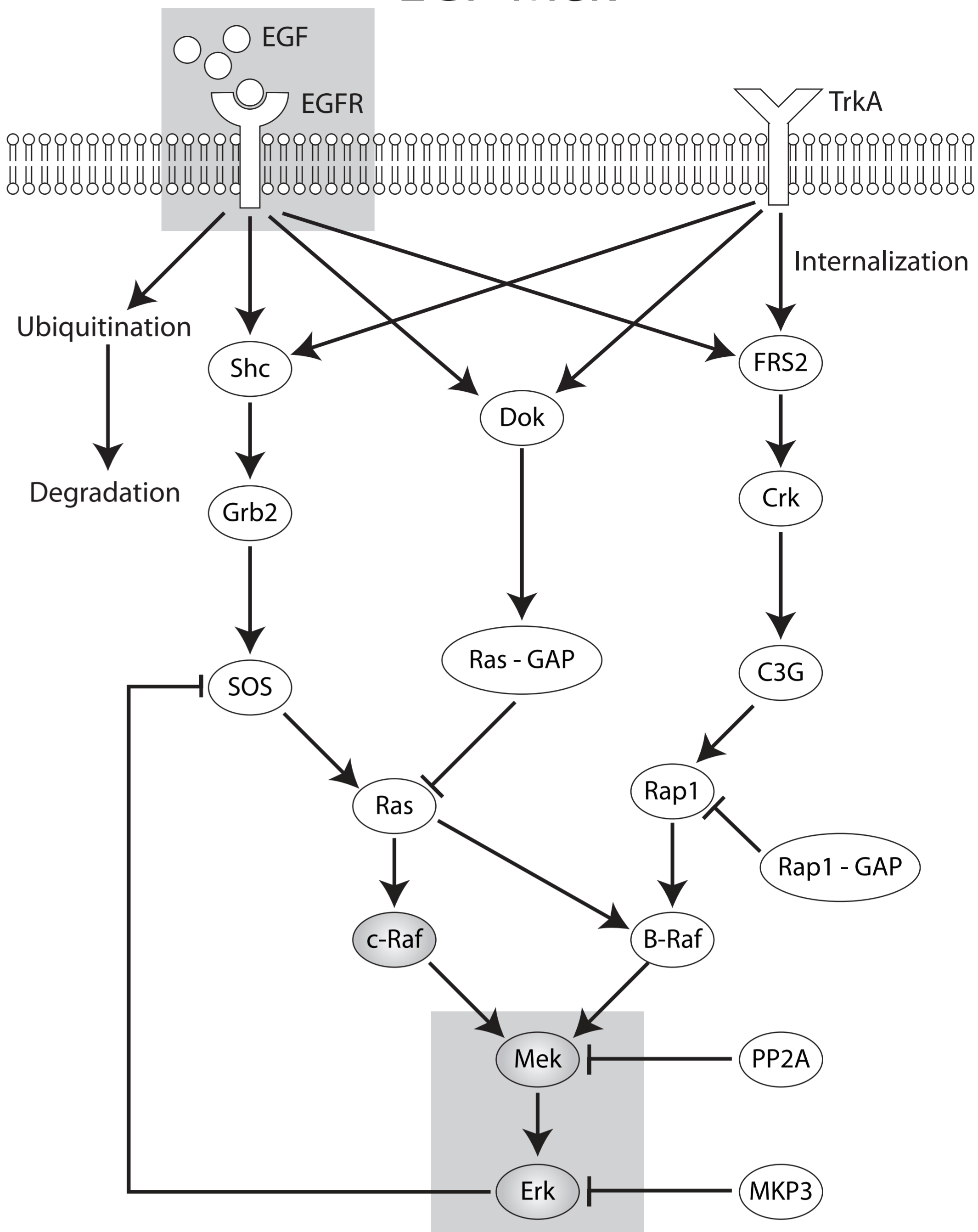**c****NGF-Mek<sup>mut</sup>**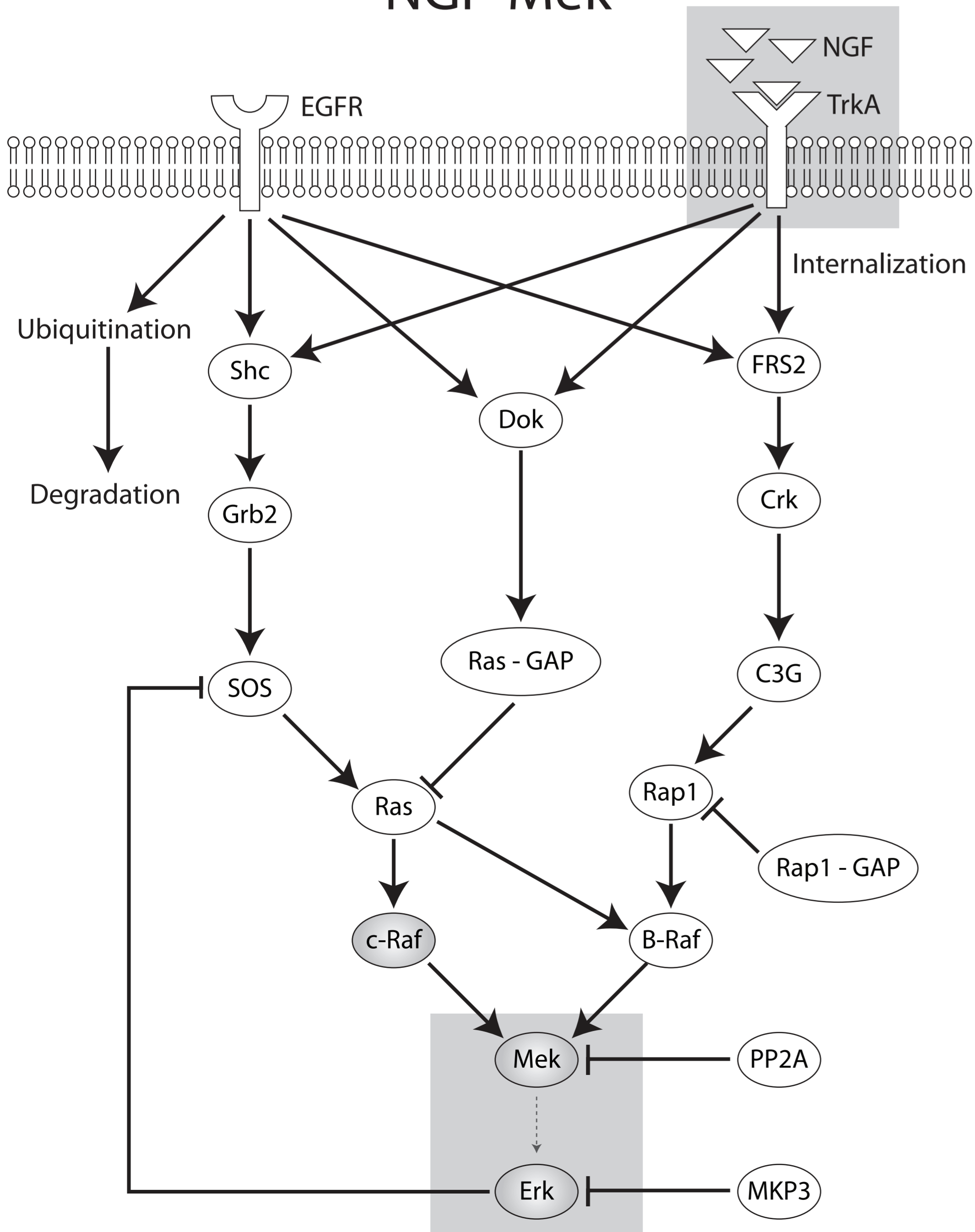**d****EGF-Mek<sup>mut</sup>**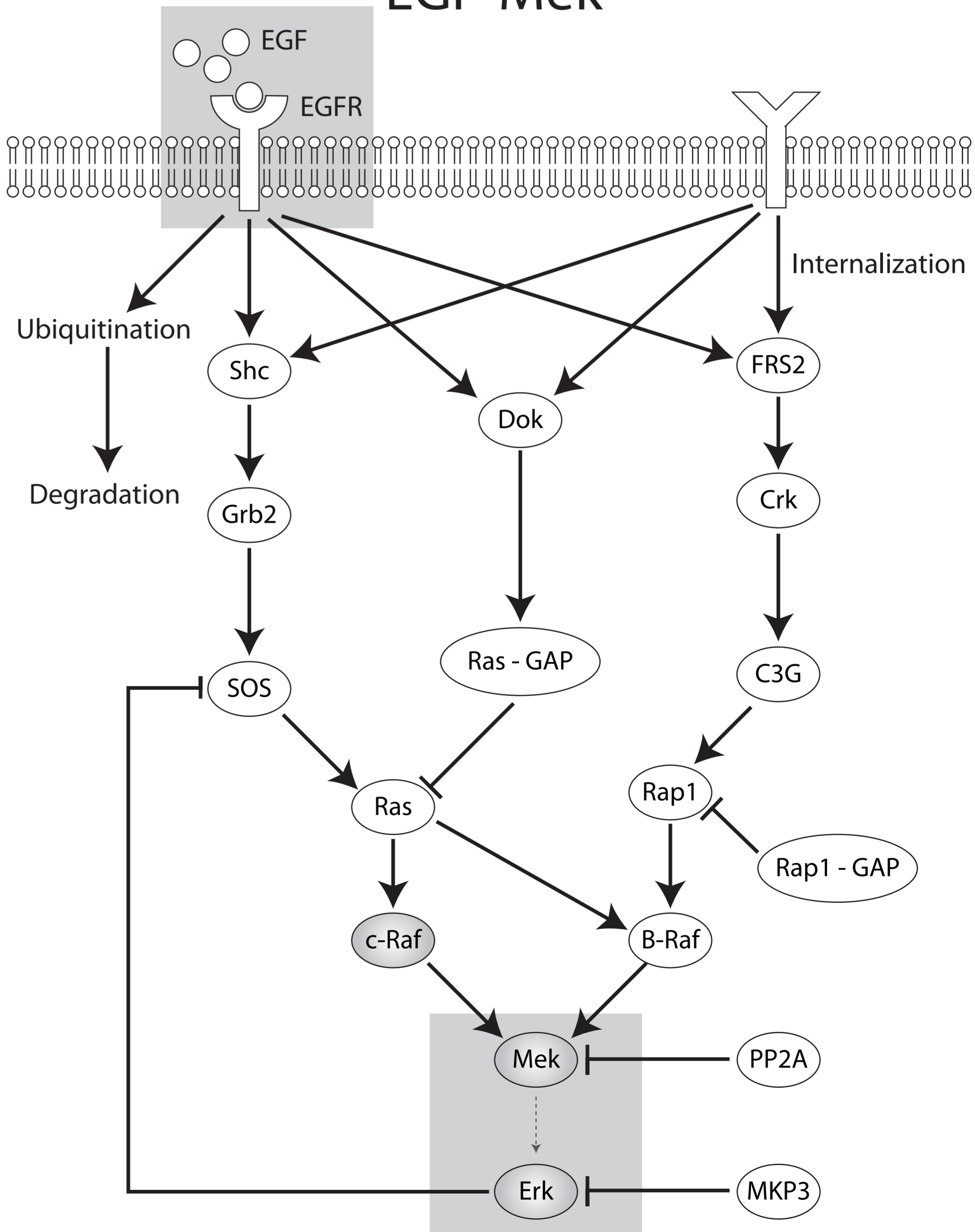

Supplement: Additional file 1 — The four distinct simulated topologies of the EGF and NGF signaling network used in Fig. 4. (a) NGF-Mek wt: the wild-type network (see Methods) with NGF stimulation. (b) EGF-Mek wt: as in (a) but with EGF stimulation. (c) NGF-Mek mut: the wild-type network with NGF stimulation, beside that here the SBML model parameter corresponding to the k cat of Mek (J136) is altered from its wild-type value (k cat = 0.15 s −1) to a value depicting a mutant Mek with a lower activity (k cat = 0.015 s −1), as indicates the thinner arrow from Mek to Erk. (d) EGF-Mek mut: as in (c) but with EGF stimulation. [file 12918_2015_170_MOESM1_ESM.pdf]

**a**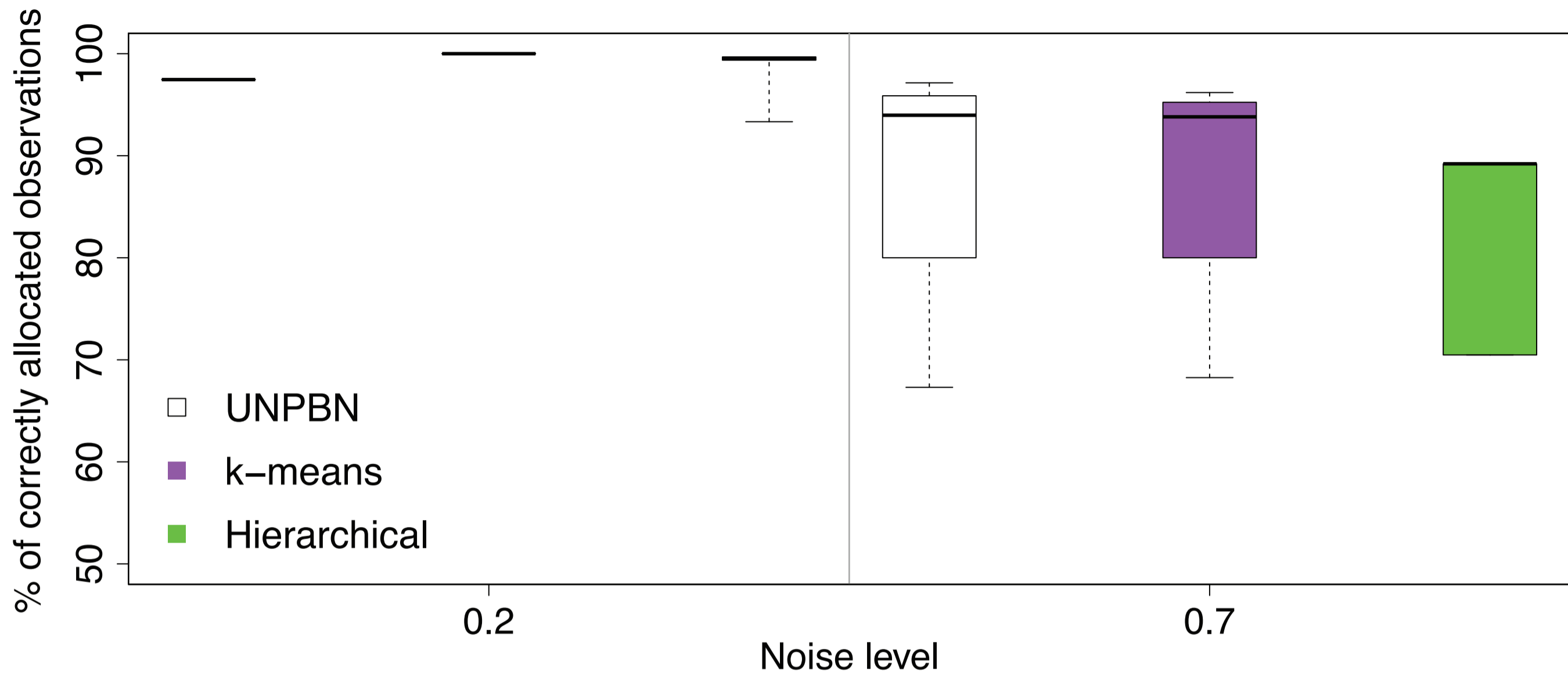**b**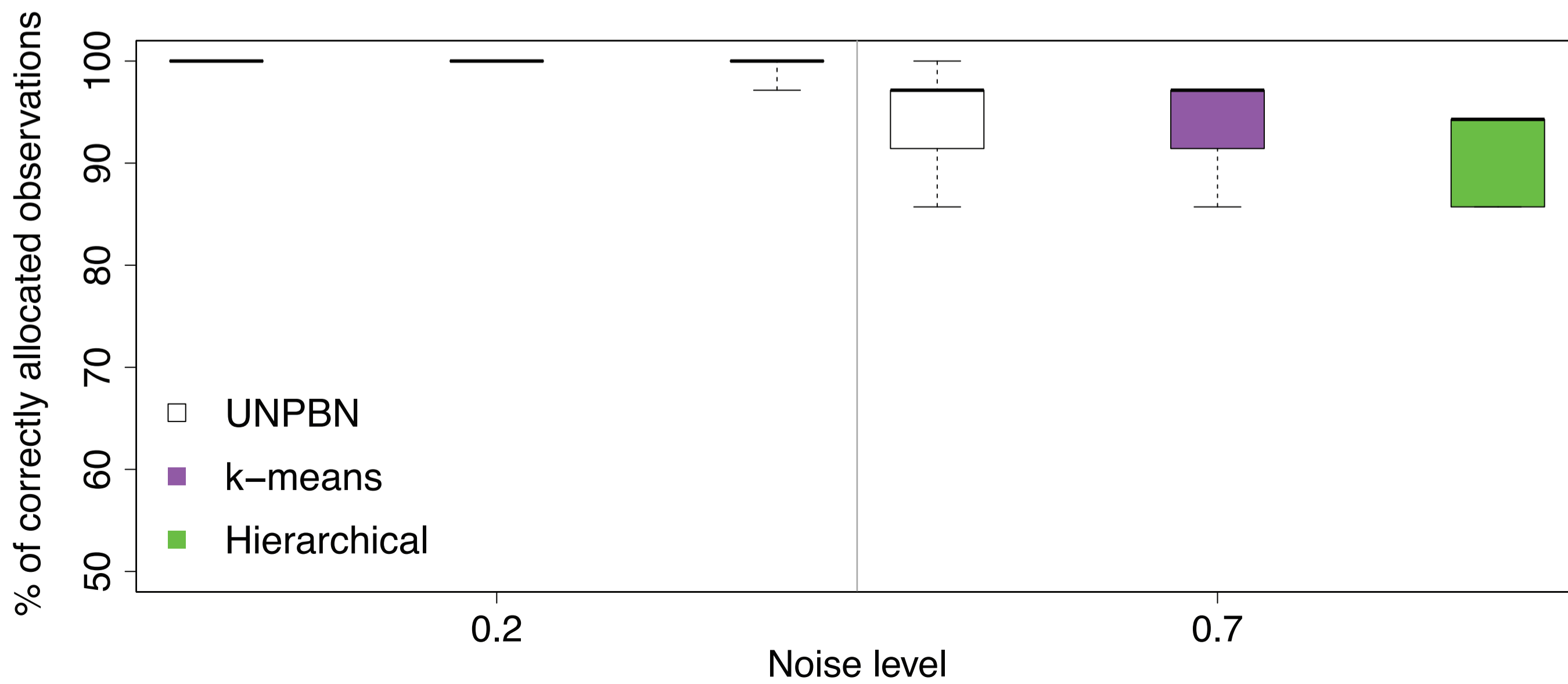

Supplement: Additional file 2 — Unmixing observations of cell subpopulations, mixed in a 1:9 ratio, by UNPBN in comparison to clustering approaches. Mixtures of observations of EGF stimulated cells (90 %) and NGF stimulated cells (10 %) were generated with noise levels of 0.2 and 0.7. Observations were sampled at one-minute intervals for 10 minutes after stimulation. For each noise level and sampled time point, observations were unmixed using UNPBN, k-means clustering (with k=2) and hierarchical clustering (taking the final two clusters). The percentages of correctly allocated observations, averaged over all time points, are indicated by boxplots for the different methods for both noise levels (line within the box, the median; box, the 0.25 and 0.75 quartiles; whiskers, the largest and smallest data points which are still within the interval of 1.5 times the interquartile range from the box). (a) The percentages of correctly allocated observations of the EGF-stimulated subpopulation. (b) The percentages of correctly allocated observations of the NGF-stimulated subpopulation. [file 12918_2015_170_MOESM2_ESM.pdf]

**a**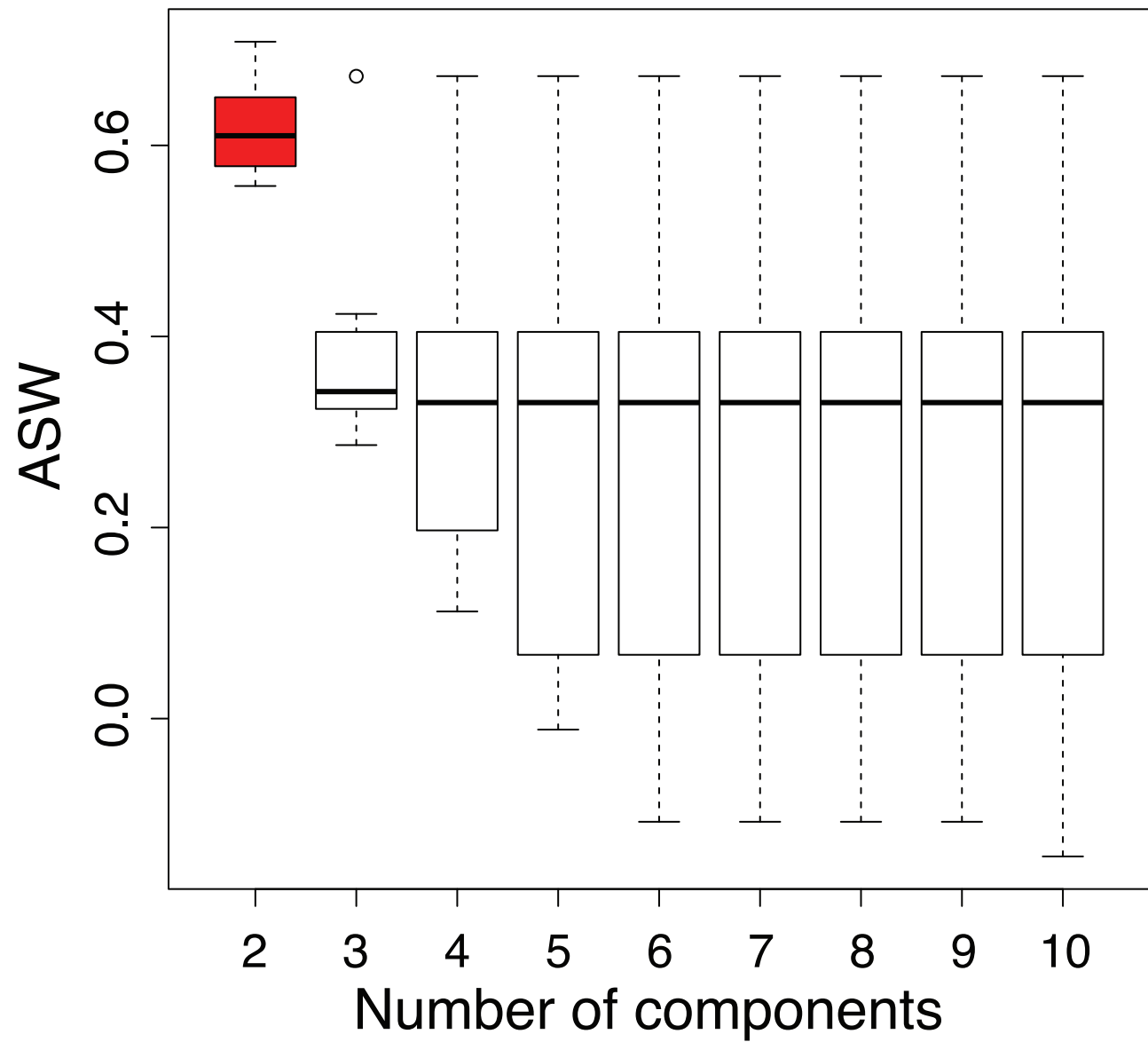**b**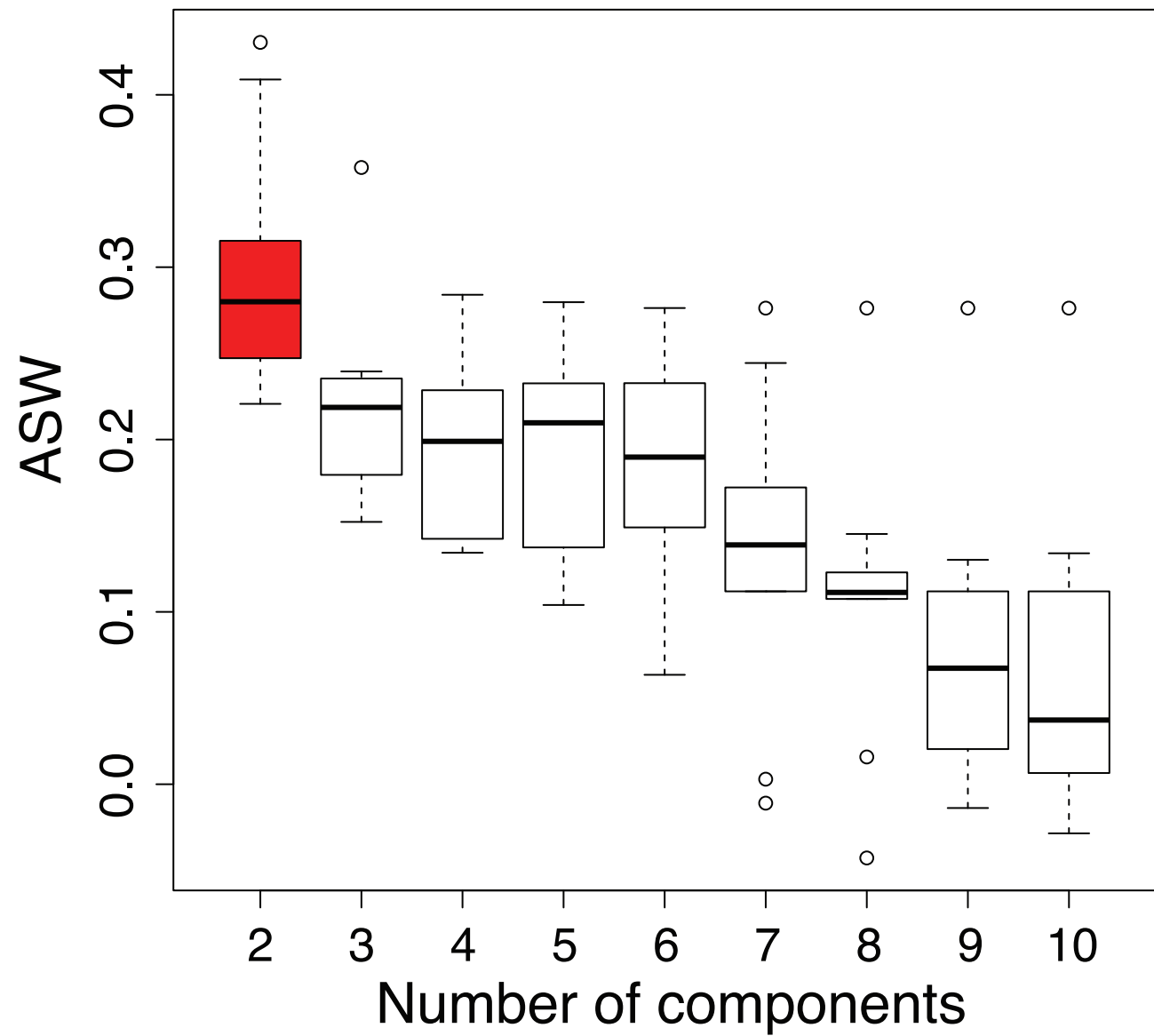

Supplement: Additional file 3 — The success of UNPBN in identifying the correct number of cell subpopulations (i.e. components) mixed in a 1:9 ratio. Mixtures of observations of EGF stimulated cells (90 %) and NGF stimulated cells (10 %) were generated with noise levels of 0.2 and 0.7. Observations were sampled at one-minute intervals for 10 minutes after stimulation. (a) A boxplot showing the ASW versus the tested number of components obtained by UNBPN analysis (here constrained in the postprocessing step to the imposed number of components). Each boxplot indicates the median (line within the box), the 0.25 and 0.75 quartiles (box), margined by the largest and smallest data points which are still within the interval of 1.5 times the interquartile range from the box (whiskers), and the outliers (dots) obtained from pooled values over all time points with a noise level of 0.2. (b) The same as (a) but with a noise level of 0.7. [file 12918_2015_170_MOESM3_ESM.pdf]
